# Text Materialities, Affordances, and the Embodied Turn in the Study of Reading

**DOI:** 10.3389/fpsyg.2022.827058

**Published:** 2022-03-14

**Authors:** Terje Hillesund, Theresa Schilhab, Anne Mangen

**Affiliations:** ^1^Department of Media and Social Sciences, University of Stavanger, Stavanger, Norway; ^2^Center for Future Technologies, Culture and Learning, Danish School of Education, University of Aarhus, Aarhus, Denmark; ^3^Norwegian Reading Centre, University of Stavanger, Stavanger, Norway

**Keywords:** digital reading, digital text, text materiality, embodied reading, embodied cognition, affordance theory, reading research

## Abstract

Digital texts have for decades been a challenge for reading research, creating a range of questions about reading and a need for new theories and concepts. In this paper, we focus on materialities of texts and suggest an embodied, enacted, and extended approach to the research on digital reading. We refer to findings showing that cognitive activities in reading are grounded in bodily and social experiences, and we explore the cognitive role of the body in reading, claiming that–influenced by tacit knowledge and the task at hand–textual meaning is enacted through a mental *and* physical engagement with text. Further, applying the concept of affordances, we examine how digital technologies have induced new ways of physically handling and mentally interpreting text, indicating that brain, body, text, and technologies are integrated parts of an extended process of reading. The aim of the paper is to encourage empirical research on the interplay between body (including brain), text, and text materialities, a focus we argue will deepen our understand of the current transformation of reading.

## Introduction

During the last decades we increasingly are reading on various types of digital devices (PCs, laptops, tablets, and smartphones) instead of reading printed matter (Balling et al., 2019). Given this development, differences between uses of printed paper and screens in reading comprehension have been the topic of a large number of empirical studies. Recent meta-analyses have added substance to claims of a paper advantage for some kinds of reading (Delgado et al., 2018; Clinton, 2019), and Delgado et al. (2018) even found that the advantage of paper-based reading had *increased* during the period 2000–2017, undermining the view that digital natives display superior screen performance.

These findings have made it urgent to better understand relations between the new multitude of text-technologies and cognitive outcomes of reading. In this theoretical paper, we will widen the perspective of the one-way causal thinking underpinning most analyses in current research (Coiro, 2020) to a more systemic approach, or to what researchers have labelled an “embodied turn” in reading research (Trasmundi et al., 2021). More specifically, we will explore reading as a process of multisensory human-technology interaction (Mangen and van der Weel, 2016), and use perspectives from embodied cognition science (Varela et al., 1991; Lakoff and Johnson, 1999; Gallagher, 2005; Clark, 2008; Rowlands, 2010; Anderson, 2014). We will introduce the concept of affordance (Gibson, 1979; Noë, 2004) and embodied reading (Glenberg et al., 2007; Glenberg, 2015), and argue for the value of these concepts in research on the current transformations of reading.

The idea of studying reading as an embodied activity seems to be nurtured by two concurrent trends: the development of new reading technologies and research on human cognition as embodied, enacted, and extended (Rowlands, 2010; Kovač and van der Weel, 2018). At the beginning of the 21st century, wirelessly connected handheld devices have facilitated a wide range of new uses of text, and for each new technology, new ways of physically handling text have evolved, from the use of keyboard and mouse to finger navigation on touchscreens. New materialities of texts have thus conditioned new ways of reading (Hillesund, 2010; Schilhab et al., 2018).

A main point within embodied cognition theory is that perception and cognition is influenced and partially constituted by the body’s engagement with the environment (Varela et al., 1991; Lakoff and Johnson, 1999; Gallagher, 2005; Clark, 2008; Anderson, 2014). Research on the theory’s hypotheses is a cross-disciplinary endeavour with a diversity of methodological approaches and theoretical nuances (Kiverstein and Clark, 2009; Walter, 2010; Malafouris, 2013; Anderson, 2014). In this paper, will concentrate on the cognitive process of reading and relate four interrelated aspects of reading and corresponding levels of analysis to various embodiment theories. This is very much in line with Coiro (2020) who recommends “a multifaceted heuristics to characterise the spectrum of digital reading experiences” (p. 19).

First, we consider reading as an act of mentally interpreting verbal text and refer to research showing that cognitive activities in this act of reading are grounded in bodily experiences and involve neural circuits associated with emotion and sensorimotor areas in the brain (Hauk et al., 2004; Aziz-Zadeh et al., 2006; Buccino et al., 2016; Desai et al., 2016; Schaller et al., 2017). Second, we will look at reading as a corporal activity and examine the cognitive role of the body in reading (Mangen, 2008; Hillesund, 2010; McLaughlin, 2015). We will emphasise the interplay of mental and physical skills in reading.

Third, we will regard reading as human action with a purpose, whether it is to be entertained from a novel, learn from a textbook, or pick up a piece of news. At a meta-cognitive level, readers bring different mental attitudes and physical reading strategies to texts depending on task as well as on technology (Ackerman and Goldsmith, 2011). At this level, we will claim that meaning of text is enacted, which means that meaning making is the result of intentional readers’ active engagements with texts and technologies. We will also relate the act of reading to the materiality of reading devices and, through the concept of affordances (Gibson, 1979; Noë, 2004; Glenberg, 2015), examine how different reading technologies allows for different ways of holding, handling, perceiving, and interpreting text (Mangen, 2008; Hillesund, 2010). Fourth, as a background aspect, we will point to the importance of the social and cultural context in which the reading activity is situated.

## The Embodied Turn

### Reading Brains

In neuroscience, brain imaging research has shown a close relationship between cognitive processes and sensorimotor and emotion areas of the brain, and, according to Lakoff and Johnson (1999), the same neural and cognitive mechanisms that allow us to perceive and move around in the world also create our conceptual systems. Gallese and Lakoff (2005) refer to evidence showing that the brain’s sensorimotor circuitry is required for understanding of concepts. For the concept of *grasping*, for instance, this means that neural circuits that form functional clusters for grasping are active not only when we physically grasp, but also when we hear and understand sentences involving the concept of *grasping* (ibid.). Pulvermüller (2005) found that corresponding sensorimotor areas were activated when participants heard utterances containing action verbs like *bite* (mouth), *kick* (feet), and *grasp* (hand). Sensorimotor areas also respond to visual and auditory information about the graspable objects (Aziz-Zadeh and Damasio, 2008; Desai et al., 2016) and to some classes of abstract concepts (Buccino et al., 2016; Schaller et al., 2017).

Importantly, research shows that sensorimotor responses are elicited even when participants *read* phrases relating to actions (Hauk et al., 2004; Aziz-Zadeh et al., 2006). Higher-level cognition, as in comprehension of narratives, relies partly on the re-enactment of perceptual and motor-sensory experiences (Engelen et al., 2011), and Rose and Dalton (2009) have found that the extended limbic region helps us prioritise and value what we read. Hence, comprehension of text is accompanied by neural activity that to a certain extent *simulates* activities occurring during concrete interactions (Glenberg et al., 2007; Schilhab, 2015, 2018). Here, the notion of “simulation” refers to the finding that motor processing of particular actions and the processing of their semantic referents are sustained by overlapping neural correlates (Barsalou, 2009; Klomberg et al., 2022). Examples of this effect have been suggested in behavioural studies of subjects reading action sentences and subsequently performing a matching or mismatching movement (Glenberg and Kaschak, 2002), in studies where participants were to assess the matching of the sentences they read with target objects presented afterwards (Holt and Beilock, 2006), and in fMRI studies of hockey players listening to hockey action sentences (Lyons et al., 2010).

According to Anderson (2007, 2010, 2014), reuse of cortical structures and neural networks is pivotal in human evolution (phylogeny) and development (ontogeny), and both language and reading networks use and connect to many parts of the brain originally evolved for other purposes. Much neuroscientific research is needed to be able to explain cognition, reading, and how the brain works, especially as the brains main task is to serve the functioning of the body and our interaction with other people and with the environment.

### Reading Bodies

Obviously, important processes are going on in the brain when we read. However, little happens before we grasp a book or a smartphone, open the book or device, and turn our attention to the text. Reading can also be described as a corporal handling of material devices, which requires a fine-tuned use of the body, primarily of the hands, fingers, and eyes, but also of head and arms (Mangen, 2008; Hillesund, 2010; McLaughlin, 2015; Baron, 2021). This physical aspect of reading highlights a different aspect of the reading process than mental interpretation, giving reading several interrelated aspects of embodiment (Mangen and Schilhab, 2012; Schilhab et al., 2018). As argued above, reading can be seen as a mental act of meaning-making that is partly grounded in a multitude of sensorimotor and social experiences. At the same time, reading can be regarded as a corporal act of physically handling reading devices and text, activities that require sensorimotor, perceptual, *and* cognitive skills. As we will see, interpretative and physical reading activities are tightly intertwined and reciprocally dependent.

All children have to learn to speak, but due to our genetic and biological dispositions, growing up in a community makes learning to speak a natural social process. In contrast, we were never born to read (Wolf, 2008). Being a recent cultural invention (starting about 5400 years ago), writing has not had time to shape our genome for reading (Dehaene and Dehaene-Lambertz, 2016). However, due to the plasticity of the brain, acquisition of literacy takes advantage of pre-existing cortical circuitries and repurpose them for written word recognition and comprehension (ibid.).

The early acquisition of literacy is an informative study of embodiment. Many children know the physicality, possibilities, and feeling of a (picture) book long before they learn to read. During reading aloud by a caretaker, the child experiences that the lines of marks can bring her to places of trolls, heroes, and heroines, and of events that evoke fear, suspense, joy, and sorrow. In the more systematic learning of literacy (usually at school), the child learns to use her vision, her hands (in pointing, scribbling, and writing), her hearing, and her voice; she learns the shapes and sounds of letters, how they combine into words and sentences, and that words and sentences have meanings. In a study of a young schoolboy’s early attempts at reading text in a picture book at home, the child, in addition to sounding out letter-by-letter, used hands and index fingers to guide his vision, eye-jumps to check for cues in the pictures and from his mother, and bursts of talk-like vocalising. These enactments he automatically judged based on felt reactions, his sensual knowledge (aisthesis), and his learned sociocultural expectations, and, actively seeking solutions, he frequently made adjustments. His early reading was a whole-body activity (Trasmundi and Cowley, 2020).

As part of the training, a child learns how to control the minute movements in her eyes, her head, and her fingers. These skills are culture-dependent and if the child is taught the western writing system, she learns that words are separated by white space, that letters and words are organised from left to right, and that her eyes must go in the same direction when reading. When she reaches the end of a line, her eyes must jump down and back in a return sweep to the exact spot where she can start reading the next line [in skilled readers this is 5–7 letter spaces from the beginning of the line (Rayner, 1998)]. When she reaches the end of the page, her vision must act again and start on top of the facing page. When that page is finished, she must turn the page and once more find the exact place to continue reading. In a fine-tuned eye-hand coordination, she must keep the body and book in positions relevant to the reading activity (McLaughlin, 2015).

Reading is thus more than an achievement of the brain. It is a multisensory and sensorimotor activity. In the embodied reading act, the oculomotor activities in the eyes and neural activities in the brain are closely intertwined and inseparable. Using eye-tracking methods, researchers have shown that the reading eye jumps along the line of text in very quick saccadic movements between short phases of fixation in which a few letters are kept in the foveal area of the eyes’ retina (Rayner, 1998). For a fluent reader, reading is fast. For a learning child, reading is slow. In the beginning, the child has to focus on every letter and spell out the meaning of every word. However, in a reciprocal influence of a learning brain, the child’s oculomotor skills improve: As she learns to use her eyes more effectively, a neural recycling process simultaneously rewires some of the visual and auditory areas in the brain for reading.

Dehaene (2009) has described this recycling process in detail. He shows how, in the reading process, incoming neural signals from the eyes are recognised in the posterior occipital lobe and increasingly directed to a left occipital-temporal area; an area that naturally is used in object and face recognition. As the child learns to read–in a striking example of neural plasticity–a part of the left occipital-temporal area takes on the new job of letter and word recognition, and through multiple bidirectional pathways, this *visual word form area* gradually connects to existing spoken language networks (ibid.). As the child learns the shapes and invariants of letters and their corresponding sounds, her reading gets faster and more automatic in a process in which the cerebellum contributes to many of the motoric skills necessary for reading (Wolf, 2008). As the child’s reading improves, her understanding of syntax increases, and “[brain] regions originally designed for other functions–particularly vision, motor, and multiple aspects of language–learn to interact with increasing speed” (Wolf, 2008, p. 126). As the decoding process gets faster, the reader “learns to integrate more metaphorical, inferential, analogical, affective […], and experiential knowledge” into the reading (ibid. p. 143). The child is on the verge of becoming an expert reader.

However, it takes years of decoding practice for children to learn to “connect meaning(s) from the text to the increasingly more complex deep reading process” (Wolf, 2017, p. 9). The physical skills needed for fluent reading also take years of practice to be fully developed (McLaughlin, 2015), and “[the visual word form area] only reaches full maturity at the beginning of adolescence–provided, of course, that the child reads regularly enough to become an expert” (Dehaene, 2009, p. 207).

According to Wolf (2017), the training pays off. When readers learn to read fluently enough to allocate more time to comprehension, a complexity of cognitive, linguistic, and affective processes opens in what Wolf describes as “deep reading”:

“Deep reading processes involve dynamic interactions among multiple processes like *imagery* and the retrieval of *background knowledge; analogical and inferential* processes that lead to *critical analysis; affective* processes like perspective-taking and empathy; and on occasion the *generative* processes leading to *insight*” (ibid., p. 9).

Wolf (2008, 2017, 2018) repeatedly underscore the importance of vocabulary and background knowledge, acquired through play and learning and stored in memory, as a prerequisite for the associative and inferential processes going on in deep reading.

### Enactment and Affordances

In a seminal article, O’Regan and Noë (2001) claim that sensorimotor action is *constitutive* of perception; we must move our hands and fingers over a surface to get a proper feeling of touch. Noë (2004) uses empirical evidence to show that our perception of the world, such as touch and seeing, is dependent on our movements and on our acting on the world. Through bodily movements, we actively change the sensory stimulation to our senses, for example, to our eyes, and there are patterns of dependency between movements of the body and sensory stimulations. These patterns of sensorimotor dependencies, O’Regan and Noë (2001) call sensorimotor contingencies, and according to O’Regan and Noë, there are two classes of sensorimotor contingencies. First, sensorimotor contingencies are determined by characteristics of the sensual apparatus. Second, they are fixed by characteristics of objects. The perception of objects is related to their shape, size, colour, texture, taste, and smell, and to their position in space, and according to Noë (2004) to the way they can be used, or to what Gibson (1979) calls their affordances, which is what objects offer the perceiver of possible actions. A pencil, for instance, can be chewed at the end, but not eaten. Both objects and properties in the environment offer opportunities to do things, but these affordances are not objective properties that can be measured in physics; they are measured relative to the animal (Gibson, 1979; Noë, 2004).

Noë (2004) claims that Gibson’s concept of affordances can be reformulated in the context of an enactive approach. To perceive is, according to the enactment view, to learn how the environment structures one’s possibility for movement and thereby to experience possibilities of actions afforded by the environment. Among these possibilities for action, reading is offered humans by texts and various text technologies. However, like Gibson (1979), Noë (2004) underscores that affordances have to be learnt, and that they are skill-relative; excellence in an activity (such as reading) “consists in the mastery of sensorimotor skills, the possession of which enables a situation to afford an opportunity for action not otherwise available” (p. 106).

In the preceding section, we saw that the cognitive and sensorimotor skills needed for fluent reading take years of practice to be fully developed, and that in the process new cortical circuits, and, according to Gallagher (2005), new motor programs are developed. Body schemas are collections of motor programs, for example, the turning of pages in a book. In page-turning, not only the anatomical parts of hands and eyes are involved, but muscle systems throughout the body are activated for purposes of maintaining balance, reading posture, and the keeping of the text in the focal area of vision (McLaughlin, 2015). In the acquisition of literacy, we develop many such body schemas. Even if some core skills are mostly the same, the use of the body, especially the hands, fingers, and eyes–and the accompanying motor programs–will differ whether you read a hardcopy novel, a textbook, or an old-fashion broadsheet newspaper, or for that matter, Facebook posts on a smartphone, an online newspaper, or a digital journal article on a laptop.

Body schemas involve motor capabilities, skills, and habits, and by saying that body schemas operate in a *close to automatic* way, Gallagher (2005) claims that “movements controlled by a body schema can be precisely shaped […] by the goal-directed behaviour of the subject” (ibid. p. 26). When we immerse ourselves in a novel, we typically concentrate on the content of the text and do not pay much attention to the fingers turning the pages or to the eyes moving along the lines; body schemas support our intentional activity, our reading. However, the body schemas and cortical structures that a reader acquires during the learning process are not arbitrary; they are constrained by biology, by social practices (the tasks), and, as we will examine further, by affordances of the reading technologies.

### Affordances of Printed Paper and of Digital Reading Devices

To be able to read at all, a text must be present for the reader’s motor and perceptual apparatus by material means, usually by ink on paper or by pixels on a screen. This dependency–or rather utilising–of intellectual tools (writings systems, books, and screens) expands our cognitive abilities, and within extended cognition theories, the tools and texts are reckoned to be an intertwined and indispensable part of the cognitive process; in this case the reading (Clark and Chalmers, 1998; Clark, 2008; Malafouris, 2013).

For centuries, printed-paper has been a main vehicle for written text. However, the last decades digital devices have become ubiquitous carriers of text. In a qualitative assessment of the Kindle e–book reader in the United States, participants responded to the look and feel of the device—its size and weight, how it fitted in the hand, the use of fingers, (un)easiness of flicking, and of navigation and screen quality (Clark et al., 2008). All these comments relate to new affordances of the device compared to a traditional printed book; at first the use of the Kindle felt unfamiliar. The participants experienced that the Kindle required different sensorimotor skills and new ways of enacting the meaning of the text.

These differences relate to varying affordances of printed books and the Kindle, and affordances are different still on an iPad or a smartphone. In a traditional book, text is printed with ink on paper and is thus stable and lasting. The paper book itself is a physical object with a certain weight, size, and shape, and the printed text is visible and tangible, and it does not disappear when you turn the pages or close the book (Mangen, 2008). On a digital display, by contrast, the physical form of the text is not tied to the surface of the medium; it is ephemeral and (in a certain way) intangible; when you click on a link in an e-book or go to a new page, the physicality of the text disappears. However, text in a digital device uses the same writing system as text in paper books, and in both cases, the text is presented on a two-dimensional surface using many of the same principles of layout. Thus, there are both similarities and differences between the affordances of digital and printed text (Hillesund and Bélisle, 2014). Affordances also vary with devices and formats (smartphones, tablets, PCs, websites, apps, books), with navigation (tap or click hyperlinks, leaf over pages), and with use of semiotic resources (verbal text, images, graphic, videos, and their configuration in multimodal and interactive text).

Used as an example, a reading student can illustrate the concepts of affordances and embodied reading. Sitting on a chair by a desk, the student may have an upright body posture with her head slightly bent forward. Both her hands are free, and holding a pen or pencil in her hand, she actively annotates a printout. While reading, the student underlines sequences of the text, she makes comments in the margins, or she takes notes on a separate piece of paper. If she is doing research for a paper, she may spread printouts and books on her desk for easy access. Much of her body is active in the reading process, and her body schemas and neural circuits differ substantially from those of her reading for pleasure at home on the couch.

Several studies show a persistent preference for print by students for some types of reading (Baron et al., 2017; Baron, 2021), even among younger readers of the digital native generation (Meishar-Tal and Shonfeld, 2018). This is partly due to a cherished affordance of printed-paper: its over-writability. Based on their study of students, Baron et al. (2017) suggest that highlighting, underling and annotations are helpful in memorising and in retrieval of important parts of articles and books. However, the habit of annotation may be more deep-rooted (Hillesund, 2010; Baron, 2021). In interviews with expert readers (academics, researchers), Hillesund (2010) found that the participants regarded the use of a pen as an aid in comprehending a text. The annotation and underlining process was a way of directing and slowing down the pace of reading to make space for reflection and inference, and to think further than the text itself. Annotation was an integral part of their scholarly reading, and the pen almost felt like an extension of themselves, indispensable in bringing forth the full meaning of the text (ibid.). Reading is thus a joint activity of body and brain, and the meaning of the text, depending on task, is enacted in an exploration of the text. Wolf and Barzillai (2009) call it an active construction of meaning, in which the reader “grapple with the text, and apply their earlier knowledge as they question, analyse, and probe.” From an extended cognition point of view, the active body, the pencil, the text, and the device are parts of an extended cognitive process (Clark and Chalmers, 1998; Noë, 2004; Malafouris, 2013).

In the example with the reading student, however, the picture would not be complete without a laptop or desktop computer on her desk, and also a smartphone, all devices with different affordances and opportunities for action. For the student, the computer offers a wealth of possibilities, or to cite Wolf and Barzillai (2009, p. 35):

“With digital text, the potential for creativity, learning, and discovery that encourage deep thought is immense. For example, interest in a Shakespearian play can drive a discovery process that links the reader not only to the text of the play and various comprehension supports, but also to relevant historical information, videos of the play, discussion groups, articles from noted literary critics, and artistic interpretations that may drive deeper reflections.”

Most learning (and other) digital activities require some sort of reading, and usually the computer system offers conditions sufficient for the reading task, whether it is searching, skimming a text for overview, or reading snippets of short texts. There are huge diversities in reading forms, and affordances must in each case be studied relative to format, device, genre, and task. Many researchers warn against a simple print/digital dichotomy (Hillesund, 2010; Clowes, 2019; Coiro, 2020; Trasmundi et al., 2021). As Clowes (2019) points out, reading must be viewed in the context of new cognitive ecologies that incorporate both screen and paper and many kinds of reading.

Nevertheless, a discussion pertaining to conditions for reading of longform digital texts has been a central theme in reading research. One of the questions of longform reading relates to ergonomic affordances of digital reading technologies. Early researchers on digital reading expressed great concern about the poor quality of computer displays. However, over the years, screen quality has improved substantially, and recent history has shown that many digital devices offer good ergonomic conditions for sustained longform reading (Kretzschmar et al., 2013).

Other affordances relate to haptic possibilities of digital reading devices. Uses of eyes, hands and fingers vary with devices, with the interface of applications, and with text formats. The materiality of reading devices and nature of navigation may in subtle ways influence the feel and flow of reading and the comprehension of text (Mangen, 2008). In a study comparing longform literary reading on a Kindle and in a pocketbook, participants performed equally well on most of the tasks. However, on measures related to their ability to reconstruct the temporality and order of events, those who had read the text in a print pocketbook outperformed the Kindle group, which is probably explained by the richer kinaesthetic and sensual experience afforded by the pocketbook (Mangen et al., 2019). It is a different experience to read a longform text (such as a novel) in a printed book, on a Kindle, or on a stationary PC-screen.

Another question is that of semiotic affordances. Semiotic modes, such as gestures, sound, speech, photographs, moving images, and writing, afford different expressions and actions and facilitate different forms of communication (Bezemer and Kress, 2016; Ledin and Machin, 2018). In multimodal expressions, typical of many digital apps, images and graphics easily attract attention and have a profound influence on how the eye navigate and read text on a webpage or on a computer screen. From the point view of sustained reading, conspicuous visual elements inevitably disturb the reading flow and make continuous reading of longform text cognitively very hard (Mangen, 2008).

In addition to being eye-catching, highlighted words and visual elements are often parts of a hypertext structure. The possibilities given by hypertext structures are one of the most useful and defining affordances of digital technology. However, affordances that are strengths in one domain may be weaknesses in another, and hyperlinks very often distract continuous and concentrated longform reading. Based on theories of attention, Mangen (2008) argues the near impossibility of being immersed in online reading in the same way we may get lost in printed books or in e-books on dedicated reading devices (or even in reading-apps on tablets and mobiles). In general, multi-layered and window-based screen environments, Web sites, apps, and ubiquitous connectivity provide an abundance of attention–switching possibilities and promise new stimuli in the form of tabs, links, hotspots, pictures, and videos. When we have options to rekindle our attention through outside stimuli, we are psycho-biologically inclined to resort to these options. It requires less mental energy to touch the screen and rekindle our attention than to resist distractions and continue reading (ibid.). Frequent attention switches are also supported by the reward-systems of the brain (Firth et al., 2019).

Another useful affordance of connected digital devices is their multi-functionality, which can also be detrimental for continuous reading (Hillesund, 2010). Heavy multi-tasking now seems to be normal, but it is proved to be diminishing our capacity for prolonged attention (Firth et al., 2019). From all sides, longform reading meets competition. At work there are always other digital tasks waiting, and in the spare time, computers offer all kinds of entertainment, from games and YouTube videos, to music, series, and TV-shows. The search function of Google is also one of the most popular, advantageous, and distractive options on the Web. However, the greatest distractions for digital longform reading are arguably the communicative affordances of social media. Most digital devices give notifications of incoming e-mails, Facebook updates, and news-items. Young people have in addition Instagram, Snap Chat, TikTok, and a variety of other apps. Not only do notifications themselves break into and disturb ongoing activities, such as reading, we are also psychologically inclined to check these notifications (Firth et al., 2019). Many users also write e-mails themselves, or they post updates and send selfies. Posting messages evokes a need to check for responses. All this electronic socialising takes time and attention that could otherwise been used for sustained reading.

## Discussion

### Digital Reading and Changes of the Brain

Given the affordances of digital technologies, it is not surprising that “screen–based reading behaviour is characterised by more time spent on browsing and scanning, keyword spotting, one–time reading, non-linear reading, and reading more selectively, while less time is spent on in–depth reading, and concentrated reading” (Liu, 2005, p. 700). This kind of reading has been labelled power browsing, bouncing, squirrelling (Nicholas et al., 2008), shallow reading (Carr, 2010), and prowling (Baron, 2015). Commentators and researchers have uttered concern that browsing and shallow reading will fundamentally change our reading behaviour, rewire our brains, and foster shallow thinking (Wolf, 2008, 2017, 2018; Wolf and Barzillai, 2009; Carr, 2010; Greenfield, 2015). The concerns seem to be supported by a recent state-of-the-art review on the possible effects of Internet use. Based on observed online behaviour, research strongly indicate that current spread and dependencies of wearables (smartphones) and the extended use of the Internet interact with shorter attention spans, less ability to fight off distractions, increased dependency on Internet as a source of factual knowledge, and less recruitment of the brain for storing information (Firth et al., 2019), all tendencies that speak against deep reading and deep thinking.

The above claims are nevertheless controversial, especially the implied assertion that an increase in shallow online reading practices has a harmful effect on expert reading skills. Clowes (2019) claims this concern is based on a specific view on how neural plasticity works. Dehaene (2009) and Dehaene and Dehaene-Lambertz (2016) suggest that recycling of neural circuits to new cognitive uses can lead to permanent loss of other cognitive abilities, but they admit that this hypothesis is built on speculation. It is nevertheless a view that underpin many of the worries concerning the detrimental effect digital devices can have on deep reading.

An alternative view is presented by Anderson (2007, 2010), who from the perspective of embodied cognition science and his neural reuse theory, claims it is “common for neural circuits established for one purpose to be exapted (exploited, recycled, redeployed) during evolution or normal development, and to be put to new uses, often without losing their original functions” (2010, p. 245). He presents empirical evidence showing that the same brain areas and circuits perform tasks in many different cognitive functions, and that one function may use many areas and circuits (Anderson, 2014). However, he does not completely rule out the possibility that original cognitive functions are affected by the learning of new ones, especially in cases with significant differences between the original and the new functions (Anderson, 2010).

According to Clowes (2019), the latter is not the case with different forms of reading, which are all very similar and use many of the same circuits and areas of the brain, such as the *visual word form area*. He claims it is unlikely that increases in some forms of digital reading should automatically be detrimental for the capacity for deep reading. To support his argument, Clowes points to the pre-digital era, in which different print formats (books, magazines, and newspapers) afforded many kinds of reading, such as searching, skimming, shallow—and deep reading. That the brain now is used for an increasing number of reading tasks does not inevitably make it less able to read deeply. On the contrary, digital technologies open a wealth of reading and learning possibilities, which, however, may vary with levels of background knowledge and reading skills, probably giving expert readers more advantages than less skilled readers (Wolf and Barzillai, 2009; Firth et al., 2019).

Within material culture studies and book history, the relation between writing technologies, text materialities, and reading has been studied for decades, showing that reading has changed substantially through its 5000 years of history, with our inwardly, silent, and fast reading being a new and modern development (Hillesund, 2010). Chartier (1995) claimed that new digital technologies would inevitably lead to changes in intellectual techniques and in our ways of reading and interpreting text. Such changes are continuously taking place, and, to finish our student example presented above, it is now easy to imagine–or even observe–students that solely rely on digital technologies in their reading and studying. What cognitive and intellectual consequences such a shift will have, is currently uncertain, and it will also depend on a multitude of technological, cultural, and political factors, and not least on policies within education.

### Children and the Future of Deep Reading

With the current focus on neuroscience, researchers of digital reading can easily be over-focused on brain changes when claims about reading skills should also include corporal activities, reading technologies, and the way uses of technology are embedded in social and economic practices. For instance, the wiring and repurposing of the brain for new skills, such as literacy, begins in infancy and depends heavily on the material cultures and social practices in the two institutions that are crucial for children’s development: family and education (Schilhab, 2015, 2017). Even if new digital reading skills do not undermine already acquired skills, such as deep reading (Clowes, 2019), expert reading has to be learned in the first place. Especially Wolf (2017, 2018) is worried that current uses of digital technologies may have a negative impact on children’s acquisition of literacy and the possibilities of becoming expert readers. Researchers agree that being an expert reader requires years of reading and training (Wolf, 2008; Dehaene, 2009; McLaughlin, 2015). In a review article on research on early reading, Barzillai and Thomson (2018) claim that the digital shift has “triggered worry about the potential harm on children’s ability to read in a deep (and) focused manner.”

There seems to be at least three aspects to this concern. One is a general concern about children’s cognitive development. There is a consensus that children develop their understanding of the world through moving and doing, in explorative play, and in interaction with others (Burnett and Daniels, 2016). However, for many children access to TV and online devices starts very early. The proportion of technologically mediated experience has increased dramatically over direct experience of the world (Kucirkova and Radesky, 2018; Sheehy and Holliman, 2018). From the perspective of embodied cognition, the consequences of this tendency for the development of language and speech–and thus for reading and thinking–is an important question.

Second, there is the question of time and quiet. In a digital world of audio-visual entertainment, online socialising, gaming, and digital education, it is uncertain whether children get enough time and attentive practice to develop from novice readers into fluent and comprehending expert readers (Wolf, 2008; Dehaene, 2009; Barzillai and Thomson, 2018; Baron, 2021). The state-of-the-art review mentioned above states that digital distractions “seem to create a non-ideal environment for the refinement of higher cognitive functions in critical periods for children and adolescents’ brain development” and that higher frequency of Internet use by children is “linked with decreased verbal intelligence” (Firth et al., 2019, p. 126).

As a third uncertainty, childhood and adolescence is the time when the human brain is most malleable and adaptive. Will neural circuits developed as a response to excessive use of screens establish cognitive patterns and behavioural habits that suppress and overrun habits and attitudes needed for expert reading? Just now, researchers believe so. Anyway, at this stage in time we are in dire need of more knowledge of what will happen to children’s cognitive abilities and reading skills when digital technology clicks in as an indispensable part of their social life and cognitive ecology (Barzillai and Thomson, 2018; Firth et al., 2019).

## Conclusion

Digital technology is now permeating all aspects of life: our professional life, our social life, our leisure time, our educational system, and our children’s childhood. In this situation, we need research on the transformation of cognition and reading. Not one theoretical approach can answer the many questions that follow the digitisation of text. In this paper, we have suggested a cross-disciplinary approach under the umbrella of embodied cognition theory.

Lately, neuroscience has given valuable insight into the neural basis of reading and will continue to do so, especially as methods and measures improve. Empirical reading research, such as surveys and experiments, may give more valuable input. Situated real-life reading behaviour must also be studied, using phenomenological introspection, in-depth interviews, and observation. Further, analysis of affordances of reading technologies may yield valuable insight into reading behaviour, and importantly, affordances must be studied relative to active subjects and their objectives. Reading is deeply embedded in cultural and social practices and consequently, reading, so reading must also be studied at a societal level.

For the study of digital reading, however, there is a special challenge: the pace of changes. Whereas codex-based reading has a history stretching back centuries and millennia, digital reading is new. Only the last decades have seen the rise of social media, smartphones, high-resolution touchscreens, and a new world of wirelessly connected handheld devices. In such a situation, we claim that theories of embodied, enacted, and extended cognition, with their overarching view on biology, technology, and culture, are very well suited for the study of the continuous changes in reading.

## Author Contributions

All authors listed have made a substantial, direct, and intellectual contribution to the work, and approved it for publication.

## Conflict of Interest

The authors declare that the research was conducted in the absence of any commercial or financial relationships that could be construed as a potential conflict of interest.

## Publisher’s Note

All claims expressed in this article are solely those of the authors and do not necessarily represent those of their affiliated organizations, or those of the publisher, the editors and the reviewers. Any product that may be evaluated in this article, or claim that may be made by its manufacturer, is not guaranteed or endorsed by the publisher.
